# Novel variants in the *CLCN4* gene associated with syndromic X-linked intellectual disability

**DOI:** 10.3389/fneur.2023.1096969

**Published:** 2023-09-15

**Authors:** Sinan Li, Wenxin Zhang, Piao Liang, Min Zhu, Bixia Zheng, Wei Zhou, Chunli Wang, Xiaoke Zhao

**Affiliations:** ^1^Department of Rehabilitation, Children's Hospital of Nanjing Medical University, Nanjing, China; ^2^Nanjing Key Laboratory of Pediatrics, Children's Hospital of Nanjing Medical University, Nanjing, China

**Keywords:** intellectual disability, MRXSRC, *CLCN4* gene, missense variant, splice site variant

## Abstract

**Objective:**

The dysfunction of the *CLCN4* gene can lead to X-linked intellectual disability and Raynaud–Claes syndrome (MRXSRC), characterized by severe cognitive impairment and mental disorders. This study aimed to investigate the genetic defects and clinical features of Chinese children with *CLCN4* variants and explore the effect of mutant ClC-4 on the protein expression level and subcellular localization through *in vitro* experiments.

**Methods:**

A total of 401 children with intellectual disabilities were screened for genetic variability using whole-exome sequencing (WES). Clinical data, including age, sex, perinatal conditions, and environmental exposure, were collected. Cognitive, verbal, motor, and social behavioral abilities were evaluated. Candidate variants were verified using Sanger sequencing, and their pathogenicity and conservation were analyzed using *in silico* prediction tools. Protein expression and localization of mutant ClC-4 were measured using Western blotting (WB) and immunofluorescence microscopy. The impact of a splice site variant was assessed with a minigene assay.

**Results:**

Exome analysis identified five rare *CLCN4* variants in six unrelated patients with intellectual disabilities, including two recurrent heterozygous *de novo* missense variants (p.D89N and p.A555V) in three female patients, and two hemizygous missense variants (p.N141S and p.R694Q) and a splicing variant (c.1390-12T > G) that are maternally inherited in three male patients. The p.N141S variant and the splicing variant c.1390-12(T > G were novel, while p.R694Q was identified in two asymptomatic heterozygous female patients. The six children with *CLCN4* variants exhibited a neurodevelopmental spectrum disease characterized by intellectual disability (ID), delayed speech, autism spectrum disorders (ASD), microcephaly, hypertonia, and abnormal imaging findings. The minigene splicing result indicated that the c.1390-12T > G did not affect the splicing of *CLCN4* mRNA. *In vitro* experiments showed that the mutant protein level and localization of mutant protein are similar to the wild type.

**Conclusion:**

The study identified six probands with *CLCN4* gene variants associated with X-linked ID. It expanded the gene and phenotype spectrum of *CLCN4* variants. The bioinformatic analysis supported the pathogenicity of *CLCN4* variants. However, these *CLCN4* gene variants did not affect the ClC-4 expression levels and protein location, consistent with previous studies. Further investigations are necessary to investigate the pathogenetic mechanism.

## Introduction

Raynaud–Claes syndrome (MRXSRC) is a rare X-linked mental developmental disorder characterized by intellectual disability (ID) or global developmental delay (GDD), language delay, autism spectrum disorder (ASD), anxiety, hyperactivity, epilepsy, brain abnormalities, and facial dysmorphism (1–6). Although those from the male sex are typically more affected than those from the female sex, some female patients may exhibit severe neurodevelopmental deficits similar to male patients due to X-chromosome inactivation (4).

MRXSRC (OMIM, #300114) is caused by mutations in the *CLCN4* gene (OMIM, #302910), leading to ClC-4 protein dysfunction. The *CLCN4* gene was initially identified and cloned by van Slegtenhorst et al. (7) who discovered an evolutionarily conserved region (CpG island). *CLCN4* is located on the Xp22.3 region of the human chromosome, but it is autosomal in other species due to chromosomal rearrangement and X-inactivation during evolution (8). Di Nguyen et al. (9) compared the *CLCN4* gene structure across multiple species and found that the translation start codons (ATG) are present at the beginning of the third exon, while the last exon (exon 13) terminates with the poly(A) tail in all examined species. The coding sequence from exon 3 to 13, which exhibits nearly 90% sequence identity among various species, is highly conserved throughout evolution. The *CLCN4* gene encodes ClC-4, an intracellular 2Cl^−^/H^+^ transporter (7, 10, 11). ClC-4 is widely expressed in the brain tissue, particularly in cerebral cortex pyramidal neurons and the cerebellar Purkinje cell layer (12). It is a 760-amino acid transport protein with 12 predicted hydrophobic domains, sharing high sequence homology and structural similarity with other family members of voltage-gated chloride channels (ClCs).

ClCs belong to two main branches: chloride channels and anion/proton antiporters. In mammals, ClC-1, ClC-2, and ClC-Ka/Kb are chloride channels located in the plasma membrane, while ClC-3 to ClC-7 are 2Cl^−^/H^+^ exchangers found in endolysosomal membranes. The structures of ClCs are remarkably similar. Among the ClC family, ClC-1, ClC-2, and ClC-K proteins are located at the plasma membrane and distributed in different tissues. ClC-K has two highly homologous isoforms that require the β-subunit barttin. On the other hand, vesicular ClCs (ClC-3 through ClC-7) have been shown to facilitate the acidification of intracellular vesicles by shunting the H^+^-ATPase currents (11). All ClC transporters exhibit strong outward rectification although the precise molecular mechanisms underlying this phenomenon are not yet fully understood (13).

ClC-4 and ClC-3 are highly conserved homologous isoforms displaying ~80% sequence identity. They are expressed in various tissues, with strong expression observed in the brain (7, 14). It is possible that these proteins may form functional ClC-3/4 heteromeric Cl^−^/H^+^ exchangers (15–17). ClC-4 homodimers are primarily found in the endoplasmic reticulum (ER), while ClC-3 forms heterodimers with late recycling endosomes/lysosomes (17). As mentioned in previous studies, ClC-4 may play an important role in the vital functions of transepithelial transport, endocytosis, endosomal acidification, and transferrin trafficking (16, 18, 19). Moreover, ClC-4 overexpression might contribute to the increased copper loading of ceruloplasmin, osteogenesis of the osteoprogenitor cell line, cancer cell proliferation and migration, and neurite outgrowth induced by nerve growth factor (20–22). However, the precise mechanisms by which the ClC-4 protein exerts these diverse influences remain unknown.

The gating glutamate plays a crucial role in the process of proton exchange for ClC-4 (23, 24). Through electrophysiological studies in Xenopus oocytes, Palmer et al. (25) found that gain-of-function (GOF) variants (including recurrent p.D89N and p.A555V variants) exhibited increased abnormal currents at negative voltages and disturbed the negative feedback mechanism of acidic luminal pH. On the other hand, loss-of-function (LOF) variants showed a shift in voltage-dependent activation toward more positive voltages. These findings suggest an impairment in the gating process of ClC-4. Guzman et al. further demonstrated that the impaired functions of ClC-4 may be compensated for or exacerbated by additional chaperone proteins and compensatory processes (26). Therefore, more sophisticated and reliable experimental systems are necessary to explore these possibilities in the future.

According to the Human Gene Mutation Database (HGMD) and previous reports (1, 2, 4, 6, 25, 27), more than 60 different variants of the *CLCN4* gene have been described, with missense variants being the most common (Supplementary Figure 1). In this study, we screened 401 children with intellectual disability (ID) and identified five CLCN4 variants in six individuals, aiming to gain further insights into the clinical and genetic characteristics of MRXSRC.

## Materials and methods

### Patient data

We screened 401 children with ID using genetic testing conducted from 1 December 2018 to 31 October 2022. We retrospectively included patients who met the following ID criteria: patients with an intellectual disability disorder with poor or absent speech, autistic features, delayed motor delay, and certain brain imaging abnormalities. The study was approved by the Ethics Committee of the Children's Hospital of Nanjing Medical University (Nanjing, China) and was conducted according to the Helsinki Declaration. Informed consent forms were obtained from all legal guardians, and we collected clinical information, demographic data, clinical progression details, neuroimaging findings, as well as blood samples from the probands and their family members.

### DNA extraction and whole-exome sequencing

A 2 ml peripheral venous blood sample was collected and placed into disposable vacuum tubes for genetic analysis. Genomic DNA was extracted from peripheral leukocytes using a DNA isolation kit (Tiangen, Beijing, China) following standard protocols. The DNA samples were subjected to whole-exome sequencing (WES) and next-generation sequencing (a total list of 42 ID-implicated genes is displayed in Supplementary Table 1). The WES analysis was conducted by Mygenostics Medical Laboratory Co. Ltd. (Beijing, China). The experimental steps involved the use of an Illumina HiSeq 2000 platform (Bio-Rad, Hercules, CA, USA) with 2 × 100-bp paired-end reads. Variants with an allele frequency >1% were excluded from further analysis. The minor allele frequency (MAF) data were obtained from various databases, including the Genome Aggregation Database (gnomAD), 1000 Genomes MAF (Chinese), dbSNP, ExAC, and an internal MAF database. The identified variants were interpreted following the guidelines of the American College of Medical Genetics and Genomics (ACMG). To validate the predicted variants in the *CLCN4* gene, Sanger sequencing was performed. This additional sequencing technique helped confirm the presence or absence of the identified variants, as predicted by *in silico* tools.

### *In silico* analysis of the *CLCN4* variants

The *in silico* analysis of missense variants was performed using polymorphism phenotyping (PolyPhen-2), sorting intolerant from tolerant (SIFT), mutation taster, PROVEAN, and CADD. Splice site variants were predicted using MaxEntScan and dbscSNV software. Alignments of *CLCN4* and its orthologs were performed using Clustal Omega (http://www.ebi.ac.uk/Tools/msa/clustalo/).

#### Plasmid construction

The wild-type full-length human *CLCN4* cDNA (GenBank NM_001830) was synthesized by Youbio Biotechnology Company. The recombinant plasmid was inserted into pcDNA3.1-3xFlag vectors by subcloning (Vazyme, Nanjing City, China). Based on the construction of the expression plasmid of wild-type pcDNA3.1-3xFlag-C1C-4-WT, four mutants, namely pcDNA3.1-3xFlag-C1C-4-D89N, pcDNA3.1-3xFlag-C1C-4-R694Q, pcDNA3.1-3xFlag-C1C-4-A555V, and pcDNA3.1-3xFlag-C1C-4-N141S recombinant expression plasmid, were constructed using the Mut Express II Fast Mutagenesis kit (Vazyme Biotech Co., Ltd., Nanjing, China). All constructs were confirmed by bidirectional sequencing.

#### Cell culture and transfection

HEK-293T cells (human embryonic kidney cells) were transiently transfected with wild-type (WT) or mutant hClC-4. Briefly, 24 h before transfection, HEK-293T cells were seeded in six-well plates with 2 ml of DMEM in each well at 37°C in an atmosphere of 5% CO_2_. After the cells were 50–70% confluent, HEK-293T cells were transfected with purified plasmids (containing either wild-type or mutant *CLCN4*) using PolyJet^TM^ (SignaGen) by the manufacturer's protocols. The final concentration of each plasmid after transfection was 0.01 μg/μL. Transfection was conducted for 4 h with 2 μg of pcDNA3.1-3xFlag-C1C-4-D89N, pcDNA3.1-3xFlag-C1C-4-R694Q, pcDNA3.1-3xFlag- C1C-4-A555V, pcDNA3.1-3xFlag-C1C-4-N141S, and pcDNA3.1-3x Flag-WT DNA, and the cells were harvested after 24 h.

#### Western blotting

HEK-293T cells were rapidly washed with ice-cold PBS, and whole-cell lysates were generated using a lysis buffer containing protease inhibitors. The cell total proteins were extracted according to the manufacturer's instructions (Beyotime, Nanjing, China). Protein concentration was determined using a micro-BCA protein assay kit with BSA as a standard (Pierce, Thermo). Then, 20 μg of total protein was separated by 10% SDS-PAGE and transferred onto the PVDF membrane. The membranes were blocked by TBS-T (0.1% Tween 20 in TBS) containing 5% non-fat milk for 1 h at room temperature and then incubated with primary antibodies against Flag (1: 1,500; Sigma-Aldrich), GAPDH (1: 1,500; ProTech) overnight at 4°C, followed by the incubation of HRP-labeled secondary antibodies at room temperature for 1 h. Membranes were visualized by the chemiluminescence reaction. Band intensity was quantified using ImageJ software (National Institutes of Health, Bethesda, MD). Differences between groups were calculated using an unpaired *t*-test with GraphPad Prism software. All experiments were repeated at least three times, and data were presented as the mean ± SD.

#### Immunofluorescence

To determine the localization of ClC-4, HEK-293T cells were grown in 24-well culture plates containing glass slides in DMEM with 10% FCS in a 5% CO_2_ atmosphere at 37°C. The wild-type and four mutant ClC-4-3xFlag plasmid DNAs were transfected using PolyJetTM (SignaGen), according to the manufacturer's guidelines. Then, the cells were fixed with methanol for 20 min at 4°C followed by staining the cells' nuclei with DAPI (4′, 6-diamidino-2-phenylindole). The cells were rinsed with PBS and incubated with the primary mouse anti-Flag (1:400; Sigma-Aldrich, St. Louis, MO) and rabbit anti-Na^+^/K^+^-ATPase antibodies (1:400; Abcam, Cambridge, MA) overnight at 4°C. The secondary antibodies were added and incubated for 2 h at 37°C. Imaging was performed on an inverted confocal laser scanning microscope using a 63/1.4 oil immersion objective. The excitation wavelength was 488 nm for the enhanced green fluorescent protein (EGFP), 543 nm for Cy3, and 340 nm for DAPI. Moreover, the wild-type and mutant ClC-4 proteins were stained with ClC-4-specific antibodies (green) to revalidate the effect of mutant protein on subcellular localization.

## Results

### Clinical features of six probands

**Figure 1:**
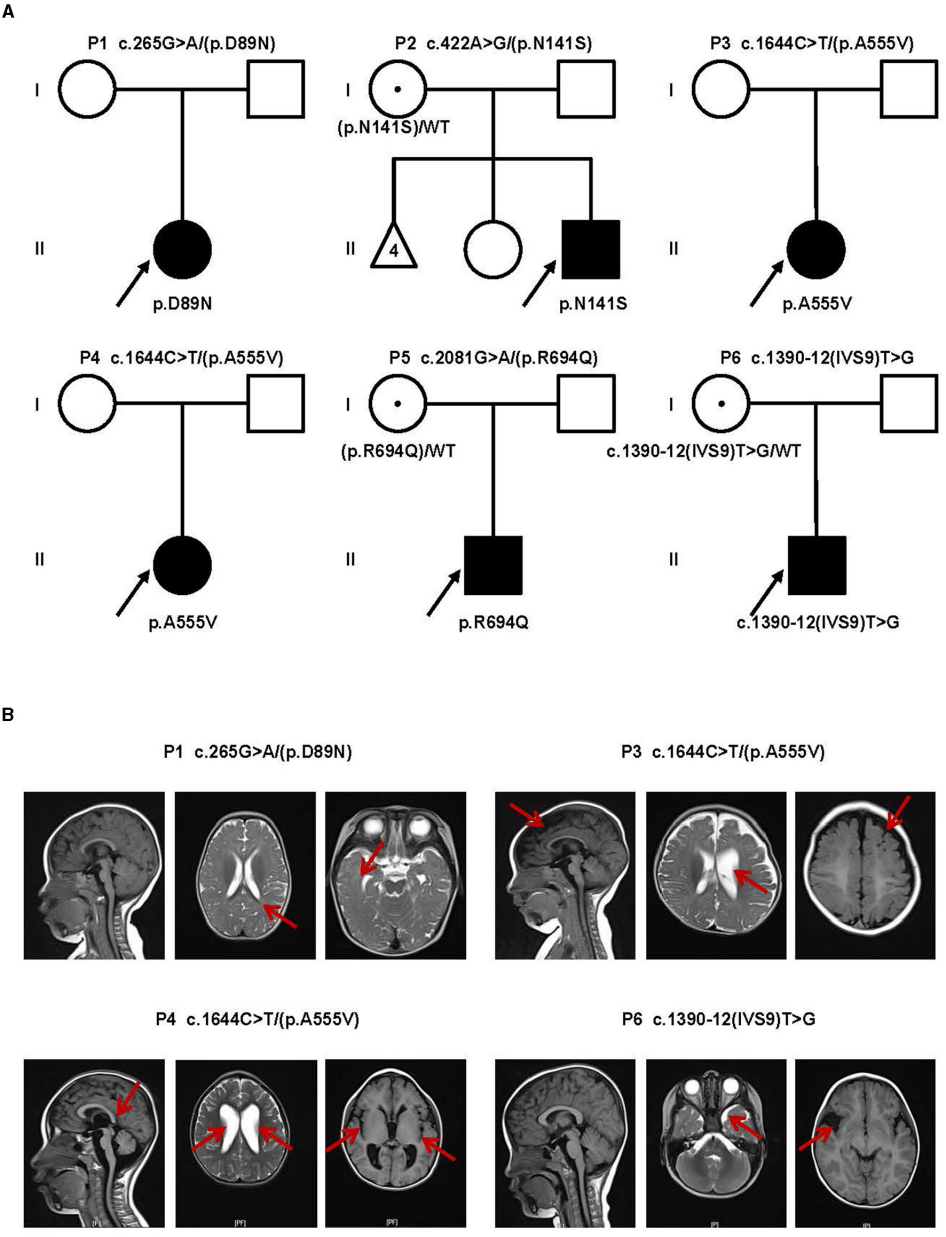
Clinical findings of affected individuals. **(A)** Pedigrees of influenced families and variants of the probands. Pedigrees of six unrelated families with *CLCN4* variants. The probands are indicated by arrows. I and II indicate the first and second generations, respectively. Squares represent male individuals, circles represent female individuals, filled symbols indicate affected individuals, open symbols indicate healthy individuals, circles with dots indicate asymptomatic carriers, and triangles represent spontaneously aborted fetuses. Number 4 represents the number of abortions. **(B)** MRI of four patients. P1: Delayed myelination. P3: A widened bilateral frontal-temporal extra brain space and an enlarged left ventricle (indicated by red arrows). P4: Brain dysplasia: small cranial volume; slightly thin posterior of the corpus callosum; widened ventricles and bilateral lateral fissure pools. P6: Possible right temporal arachnoid cyst and slightly wide left temporal subcranial plate gap.

In this study, we identified six probands (three male and three female patients) who carried rare *CLCN4* variants. MRXSRC was shown to be caused by variants in the *CLCN4* gene according to previous research (14) and the OMIM database. The pedigree of the families with *CLCN4* variants is presented in Figure 1A. All six children were below 3 years of age at the time of presentation and exhibited varying degrees of global developmental disorders and language delays. Among the probands, three girls (P1, P3, and P4) displayed microcephaly, while two boys (P2 and P5) had microcephaly below the median range. The boys (P2, P5, and P6) also exhibited autistic behaviors. The clinical phenotypes observed in these patients were complex and varied. P1 exhibited distinctive features such as microcephaly, esotropia, and hypertonia. P2 demonstrated limited eye contact, a lack of interest in social interactions, and an absence of normal imitative actions. He was also evaluated by the Autistic Behavior Checklist (ABC) (score < 50) and Developmental Screen Test (DST) [developmental quotient (DQ) score < 50 and mental index (MI) score < 50]. P3 initially showed normal development at birth but exhibited hypertonia at a physical examination conducted at 7 months old. The child's motor and cognitive function were delayed compared to other children of the same age. At 11 months, P3′s height was 68.8 cm (< -1SD), weight was 7.8 kg (< -1SD), and head circumference was 40.6 cm (< -3SD). P3 was also evaluated by the DST (DQ score < 45 and MI score < 50). P4 experienced swallowing difficulties, slight limb hypertonia, and occasional involuntary shaking although there was no history of seizures. P5 had feeding difficulty and behavioral abnormalities. Neurological examination revealed poor visual and auditory facial reflexes. P6 suffered from septicemia during infancy and was non-verbal at the age of 2 years. Further details can be found in the Supplementary material. Due to the loss of contact with patients and limited resources, we encountered challenges in obtaining complete follow-up data. Overall, the clinical features observed in these six probands were characterized by a range of developmental delays, language impairments, microcephaly, hypertonia, behavioral abnormalities, and other associated symptoms.

Brain magnetic resonance imaging (MRI) was performed in four patients (P1, P3, P4, and P6), and the results revealed a diverse spectrum of abnormalities, as shown in Figure 1B. In the case of P1, the MRI displayed delays in myelination development. For P3, the MRI revealed a widened bilateral frontal-temporal extra brain space and an enlarged left ventricle. P4 exhibited brain dysplasia, where the cranial volume was small, and the bilateral frontal areas appeared more pointed, the posterior part of the corpus callosum was slightly thin. The supratentorial ventricles were significantly widened, the bilateral ventricles were dominant, the bilateral lateral fissure pools were wide, the cerebral sulcus was slightly wide and deep, and the extracerebral space was slightly wide. P6 had a possible right temporal arachnoid cyst and a slightly wide subcranial space in the left temporal plate. Overall, the brain MRI findings in these patients demonstrated a range of abnormalities, including delays in myelination development, enlarged ventricles, brain dysplasia, and the presence of cysts. These imaging abnormalities contribute to the understanding of the underlying neurological manifestations associated with the *CLCN4* gene variants in these individuals.

### Variants detected in the *CLCN4* gene

**Figure 2:**
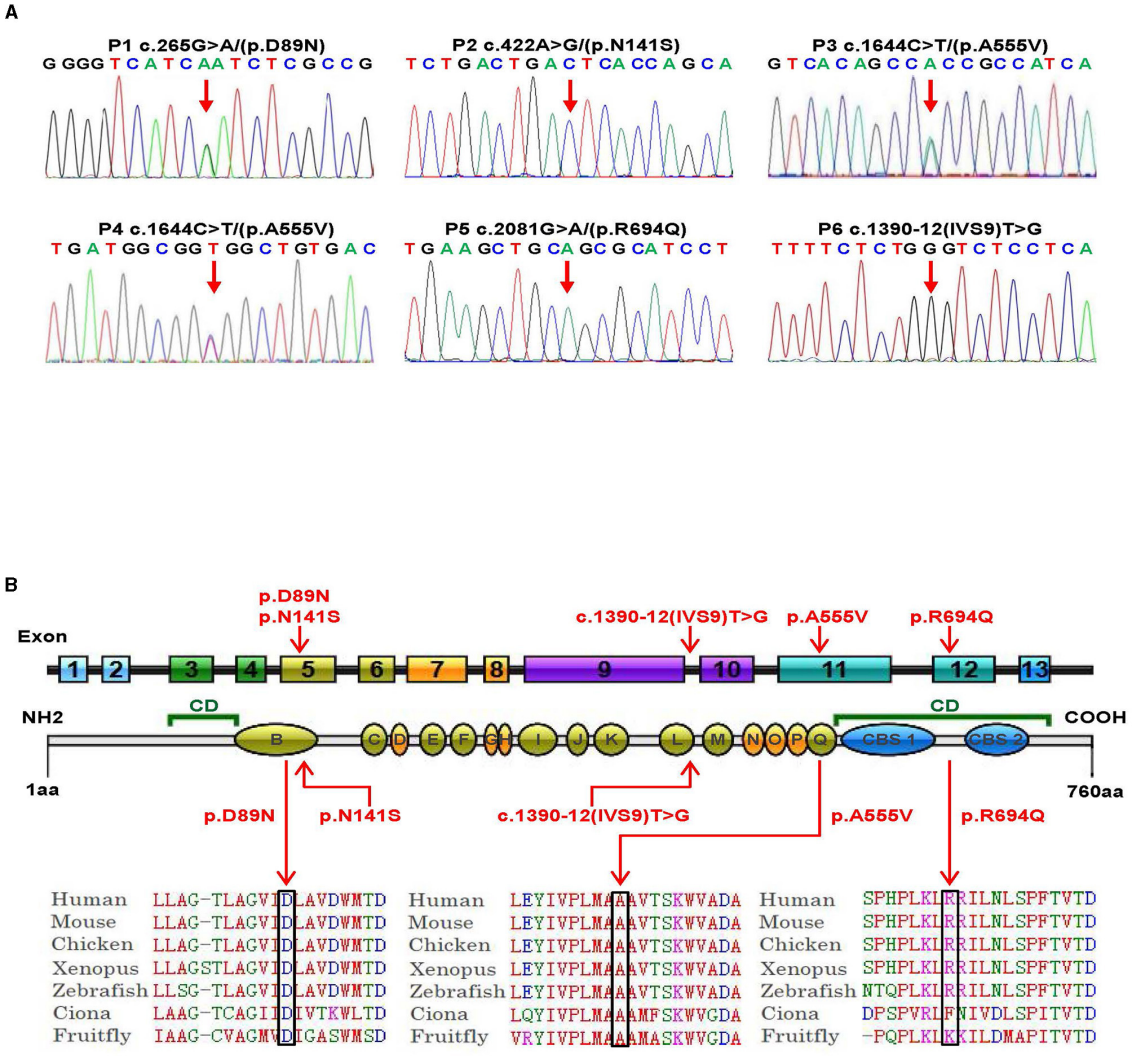
Genetic bioinformatics analysis. **(A)** Gene sequencing of the six patients with *CLCN4* variants. The probands show a nucleotide change (red arrow). Two peaks show a heterozygous nucleotide change and a single peak shows a hemizygous nucleotide change (A, adenosine; G, guanine; C, cytosine; T, thymidine). **(B)** The variants identified in this study are shown in red. B to Q represent transmembrane and intramembrane domains (green domains represent helical transmembranes and yellow domains represent helical-intramembranes). Multiple-sequence alignment confirms the evolutionary conservation of missense variants in the animal kingdom (CD, cytoplasmic domain; CBS, cystathionine-beta-synthase domains).

Genetic analysis was conducted on DNA samples from all participants using WES and Sanger sequencing of *CLCN4*. We identified four missense variants (p.D89N, p.N141S, p.A555V, and p.R694Q) and a novel splice site variant (c.1390-12T > G) in the six patients (Figure 2A). Three unrelated female patients carried two *de novo* heterozygous recurrent missense variants, p.D89N (P1) and p.A555V (P3 and P4). Three hemizygous variants that are maternally inherited were identified in three unrelated male patients: P2 carried a novel missense variant, p.N141S, P5 carried a novel splicing variant (c.1390-12T > G), and P6 carried the p.R694Q variant that had been reported in two healthy female carriers. According to the ACMG guidelines, the p.D89N, p.N141S, and p.R694Q variants were predicted to be of uncertain significance [(PM1 +PM2 + PP3), (PM2 + PP2 + BP4), and (PP2 + PP3)], while the p.A555V variant was predicted to be pathogenic (PS2 + PS4 + PM2_supporting + PP3) (13) (Table 1).

**Table 1:** Variants of the *CLCN4* gene identified in this study.

**Patient**	**Nucleotide change**	**Amino acid change**	**Location**	**Domain**	**Hom/Het**	**ACMG**	***De novo***	**Reported**
P1	c.265G > A	p.Asp89Asn	E5	TM	Het	VUS (PM1 + PM2 + PP3)	Yes	(25)
P2	c.422A > G	p.Asn141Ser	E5	TM	Hemi	VUS (PM2 + PP2 + BP4)	Mother	No
P3	c.1644C > T	p.Ala555Val	E11	TM	Het	P (PS2 + PS4 + PM2_Supporting + PP3)	Yes	(4)
P4	c.1644C > T	p.Ala555Val	E11	TM	Het	P (PS2 + PS4 + PM2_Supporting + PP3)	Yes	(4)
P5	c.2081G > A	p.Arg694Gln	E12	CD	Hemi	VUS (PP2 + PP3)	Mother	No
P6	c.1390-12(IVS9)T > G	-	IVS9	-	Hemi	VUS (PM2 + PP3)	Mother	No

CD, cytoplasmic domain; E, exon; Het, heterozygous; Hemi, hemizygous; Hom, homozygous; IVS, intron; P, pathogenic; P1-P6, Patient 1-Patient 6; TM, transmembrane domain; VUS, variants of uncertain significance. The criteria for pathogenic variants can be divided into very strong (PVS1), strong (PS1~4), moderate (PM1~6), or supporting (PP1~5). The evidence for benign variation can be classified as stand-alone (BA1), strong (BS1~4), or supporting (BP1~6). Among them, the number is only used as a class label for reference and is not meaningful.

Based on five *in silico* predictions (PolyPhen-2, SIFT, mutation taster, PROVEAN, and CADD), all variants were most likely damaging and deleterious (Supplementary Table 2). The p.D89N, p.N141S, and p.A555V variants were located in the transmembrane domain, while the p.R694Q variant was in the cytoplasmic domain (Figure 2B). These altered residues are highly evolutionarily conserved across species except for p.N141S, indicating that they are likely harmful variations (Figure 2B). A comparison of the homology of nine members of the ClCs family revealed that the p.D89N and p.A555V variants were highly conserved, whereas the p.R694Q variant was partially conserved, and the p.N141S variant was not (Supplementary Figure 2).

According to the ACMG guidelines, the novel splice site variant (c.1390-12T > G) was predicted to be of uncertain significance (PM2 + PP3). This variant is located at the 12th position of the splice acceptor site of exon 10 and has not been previously reported in any public genomic databases. *In silico* predictions using MaxEntScan and dbscSNV suggested that this splicing variant was likely deleterious.

#### *In vitro* studies of CLCN4 variants

**Figure 3:**
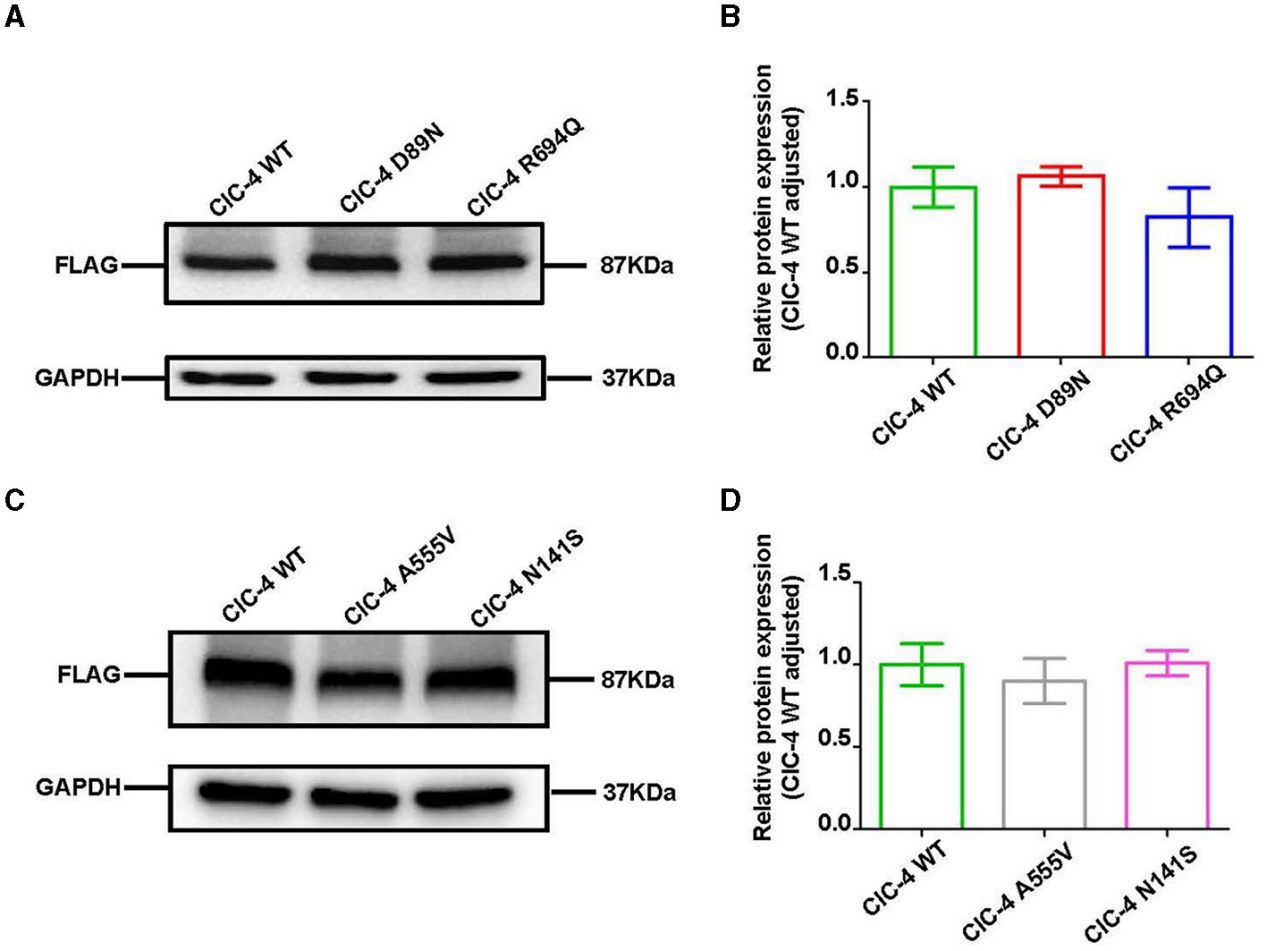
Western blot analysis of wild-type and mutated ClC-4. **(A)** The protein levels of ClC-4 WT, D89N, and R694Q variants in HEK293 cell. **(B)** Quantification analysis of the ClC-4 WT, D89N, and R694Q variants. **(C)** The protein levels of ClC-4 WT, A555V, and N141S variants in HEK293 cell. **(D)** Quantification analysis of the ClC-4 WT, A555V, and N141S variants. Western blot analysis of the HEK293T cell expressing wild-type and mutant ClC-4. The proteins are expressed at similar levels. Differences between groups were calculated using an unpaired *t*-test with GraphPad Prism software. All experiments were repeated at least three times, and data were presented as the mean ± SD. It was not statistically significant (*P* > 0.05). All lanes are taken from the same gel and blot.

**Figure 4:**
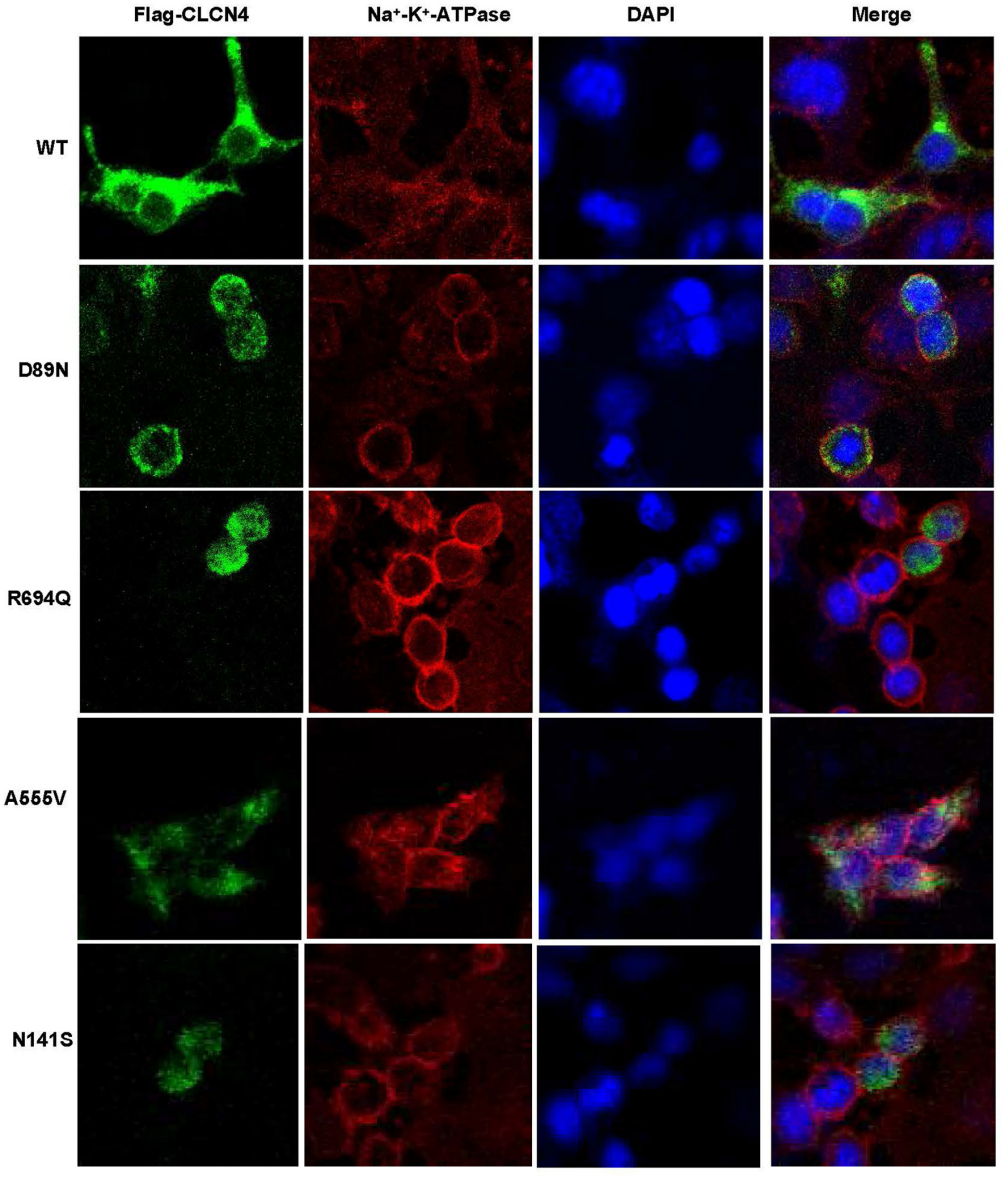
Immunofluorescence analysis of wild-type and mutated ClC-4. Subcellular localization of human ClC-4 variants expressed in HEK293T cells. ClC-4 wildtype and the four mutant proteins localize mainly to the ER. The mutant ClC-4 did not seem to alter the subcellular localization of ClC-4.

We examined the levels of expression of ClC-4 in whole cells of HEK-293T and observed that cells were transfected with wild-type and four mutant constructs (p.D89N, p.N141S, p.A555V, and p.R694Q). The flag-specific monoclonal antibody revealed a protein band (84.9 kDa) with similar protein expression levels for all constructs (Figure 3). Subsequently, an immunofluorescence assay was performed to evaluate the localization of WT-ClC-4 and Mut-ClC-4 in HEK-293T cells. The result showed that all four *CLCN4* variants do not alter the subcellular location of ClC-4 (Figure 4).

**Figure 5:**
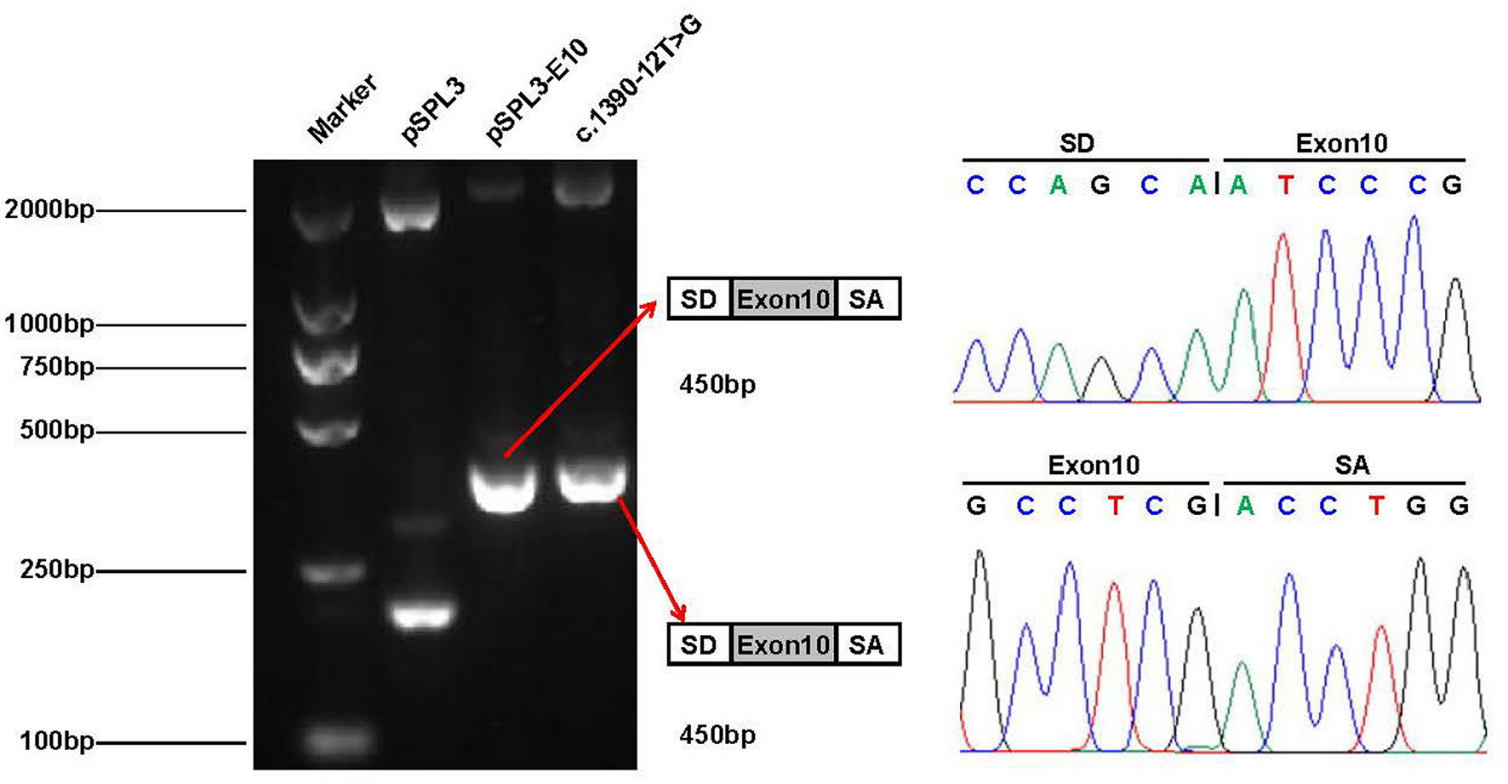
Minigene analysis of the novel splicing variant c.1390-12T > G. RT-PCR amplification for aberrant splicing transcripts, agarose gel separation, and subsequent Sanger sequencing was conducted. They were confirmed by bidirectional sequencing. RT-PCR products were analyzed by agarose gel electrophoresis, and no size variants were observed. Sanger sequencing demonstrates no skipping exon.

To verify whether the c.1390-12T > G variant impaired mRNA splicing, we performed a minigene splicing assay *in vitro*. We evaluated the effect of the variant on splicing by the pSPL3 minigene reporter in HEK-293T cells. The cDNA analysis prepared from HEK293 cells indicated that the c.1390-12T > G variant may disturb normal splicing *in vitro*. However, agarose gel electrophoresis performed by RT-PCR products showed that no skipping exons occurred (Figure 5). Finally, our minigene splicing assay confirmed that the novel variant of c.1390-12T > G did not affect the normal splicing of *CLCN4* mRNA.

## Discussion

In this study, we identified six probands from unrelated Chinese families with one splice site variant and four missense variants of the *CLCN4* gene. *CLCN4* gene mutations have been previously associated with MRXSRC (28, 29), and the clinical findings in our probands were consistent with the pathophysiology observed in MRXSRC, including a broad phenotypic spectrum ranging from ID, language delay, abnormal behaviors, brain abnormalities, and facial dysmorphism (2).

MRXSRC is an extremely genetically heterogeneous sex chromosome-associated ID, with those of the male sex being more frequently affected than those of the female sex. *CLCN4* variants have been shown to exhibit complete penetrance, with variable expressivity in those of the male sex and partial penetrance and variable expressivity in those of the female sex (5). In our study, all male patients (P2, P5, and P6) exhibited autistic features and severely delayed speech development or were non-verbal. Palmer et al. (4) reported a French family (MRX15) with non-syndromic X-linked intellectual disabilities, in which all affected male patients had mild-to-profound ID/GDD with variable behavioral problems, while two female heterozygotes were unaffected. These observations suggested that *CLCN4* variants associated with MRXSRC are sex-related. Epilepsy is also a significant characteristic feature of *CLCN4*-related neurodevelopmental conditions. Palmer et al. (25) found that over 50% of male patients and 20% of female patients had seizures. Although our patients did not experience any seizures, ongoing surveillance of the incidence of seizures in childhood would be clearly warranted. A previous study found that ID severity and neuroimaging progressively worsened in some cases, and some symptoms appeared after the age of 3 years, including behavioral problems, psychiatric disorders, and emotional disorders (4). These results suggest that more attention should be paid to managing mental health problems in older children.

The *CLCN4* gene comprises 13 exons and 12 introns and it encodes a transporter protein that comprises 760 amino acids in human beings. The ClC-4 protein contains cytosolic cystathionine-beta-synthase (CBS), transmembrane (TM), and intramembrane (IM) domains. The TM domains affect the function of ion channels, whereas CBS domains affect the gating and localization of a transmembrane protein and regulate membrane components (30). Variants in the *CLCN4* gene reduce the activity of Cl^−^/H^+^ exchange, disrupt homeostasis and intracellular transport of vesicles, weaken protein function, and affect neuronal differentiation (26).

In this study, we identified two recurrent heterozygous *de novo* missense variants in three female patients, including p.D89N in P1 and p.A555V in P3 and P4. Three male patients carried hemizygous variants that are maternally inherited, with the two missense variants (p.N141S in P2 and p.R694Q in P5) and the splice site variant (c.1390-12T > G) in P6. Variants such as p.N141S and c.1390-12T > G are novel, and p.R694Q has been identified in two asymptomatic heterozygous females in a previous study. Importantly, p.D89N, p.N141S, and p.A555V variants were located in the TM domains of the ClC-4 protein. In a previous study, GOF variants were found, namely, p.D89N and p.A555V variants, which disrupted the gating process of ClC-4 that prevents inward currents (25). Therefore, it is possible to speculate that these regions are crucial for normal protein function, which is supported by previous evidence (1).

The six children with *CLCN4* variants presented with a neurodevelopmental spectrum disease featuring intellectual disability (ID), delayed speech, autism spectrum disorders (ASD), microcephaly, hypertonia, and abnormal imaging manifestations. The onset age of our patients was under 3 years old. The genetic analysis indicated that pathogenic variants of the *CLCN4* gene, identified using multiple *in silico* prediction tools, may result in the loss of function of the ClC-4 protein. In P6, we identified a novel splice site variant c.1390-12T > G, which was predicted to lead to affect the normal splice of ClC-4 mRNA. However, the *in vitro* minigene splicing reporter assay revealed that the splice site variant had no significant effect on RNA splicing. It is possible that the *in vitro* minigene splicing experiments cannot fully simulate gene splicing *in vivo*. In *in vitro* experiments, we found that the four mutants (p.D89N, p.N141S, p.R694Q, and p.A555V) did not affect the ClC-4 protein level and localization. In the previous study, p.A555V did not affect the expression and location but reduced the ClC-4 currents (4). Multiple studies have shown that *CLCN4* mutants do not affect protein localization and expression but impact currents (1, 4, 25). It is required to conduct more complex experimental systems, such as animal models and current assay to explain the pathogenetic mechanism. Despite its certain limitations, our findings of multiple changes in a ClC-mediated Cl^−^/H^+^ shift by disease-related variants promote further studies in understanding the molecular genetic basis of X-linked ID.

Furthermore, Hu et al. showed that ClC-4 deletion of hippocampal neurons interfered with the normal progress of neuronal differentiation. The cells showed a 30% reduction in neurotic branching and outgrowth. Similar changes were observed in neurons cultured from *CLCN4* knockout mice (5). Another study suggested that among the ClC family of proteins, ClC-4 is involved in NGF-induced meson activation and neurite growth, indicating that ClC-4 plays an important role in nervous system development (31, 32). The ClC family of Cl^−^ channels and transporters form dimers with one permeation pathway in each monomer. In addition to typical homodimers, some isoforms can function as heterodimers (31). Weinert et al. (33) found that ClC-3 and ClC-4 are prone to form stable heterodimers that reside mainly on late endosomes. The researchers showed that a substantial part of ClC-4 is found physically combined with ClC-3 *in vivo*. Without ClC-4, ClC-3 can reach endosomes, while the stable presence of ClC-4 in endosomes is largely because of the help of ClC-3. In the absence of ClC-3 as an important binding partner, ClC-4 remains in the endoplasmic reticulum where it is most likely degraded (33).

To date, the recurrence rate of p.A555V variants reported in the literature is high (see Supplementary Table 3). We found that these patients seemed to have more severe language delays, microcephaly, and feeding problems (Tables 2, 3), providing direction for the treatment and home care of patients. Due to the heterogeneity of the disease, developing more effective treatment options remains challenging. Currently, most treatments are supportive and can improve patients' quality of life.

**Table 2:** Recurrent *CLCN4* variants in our study and a previous study.

**Probands**	**P3 A555V (This study)**	**P4 A555V (This study)**	**P44 A555V (4)**	**P84 A555V (25)**	**P85 A555V (25)**	**P86 A555V (25)**
Neurological features	No	Hypertonia	No	Cerebral palsy, hypotonia	Hypotonia, brisk tendon reflexes, stereotypical hand movements (chorea)	Mild hypotonia
Delayed speech	Severe	Severe	Non-verbal	Yes	Yes	Speaking in sentences (40% understandable by others)
Behavioral issues	No	No	No	No	No	No
Seizure disorders	No	No	No	No	No	No
Head circumference	< 3rd centile	< 3rd centile	< 2nd centile	< < 2nd centile	< < 2nd centile	< 3rd centile
Weight	< 3rd centile	25–50th centile	25–50th centile	< < 3rd centile	< < 3rd centile	2nd centile
Height	< 3rd centile	10–25th centile	3–10th centile	< < < 1st centile	< < < 1st centile	3rd centile
Gastrointestinal symptoms	No	Difficulty swallowing	Feeding difficulties, growth retardation/failure to thrive, gastrostomy fed, constipation	Failure to thrive, gastrostomy feeding, constipation, gastroesophageal reflux, allergic colitis	Feeding difficulties, gastroesophageal reflux	Failure to thrive
Other phenotypical features	No	Occasional involuntary shaking	No	Ichthyosis	No	No

**Table 3:** Recurrent *CLCN4* variants in our study and a previous study.

**Probands**	**P1 D89N (This study)**	**P74 D89N (25)**
Neurological features	Hypertonia	Ataxia, unsteady gait
Delayed speech	Yes	Yes (only five words at 3 years old)
Behavioral issues	No	No
Seizure disorders	No	No
Head circumference	< 3rd centile	Relative microcephaly
Weight	Normal	Not reported
Height	Normal	Not reported
Gastrointestinal symptoms	No	Difficulty swallowing, gastroesophageal reflux
Other phenotypical features	No	No

## Conclusion

In conclusion, our study reported six Chinese children with variants in the X-linked ID-related *CLCN4* gene and expanded the spectrum of *CLCN4* variants. Despite these novel findings, further expression analyses and functional studies are required to clarify the molecular mechanisms of the disease. At present, the comprehensive understanding of the pathogenic mechanism underlying MRXSRC is still unclear, and more cases need to be collected for further analysis to improve the early diagnosis, treatment, and prenatal and postnatal care of intellectual disorders and their comorbidities.

## Data availability statement

The original contributions presented in the study are publicly available. This data can be found here: Leiden Open Variation Database (LOVD), https://databases.lovd.nl/shared/individuals, Individual IDs 00424012, 00424013, 00424069, 00424070, and 00424071.

## Ethics statement

The studies involving humans were approved by Ethics Committee of the Children's Hospital of Nanjing Medical University (Nanjing, China). The studies were conducted in accordance with the local legislation and institutional requirements. Written informed consent for participation in this study was provided by the participants' legal guardians/next of kin. Ethical approval was not required for the studies on animals in accordance with the local legislation and institutional requirements because only commercially available established cell lines were used. Written informed consent was obtained from the individual(s), and minor(s)' legal guardian/next of kin, for the publication of any potentially identifiable images or data included in this article.

## Author contributions

XZ and CW designed the study. SL drafted the article and performed the experiments. CW and SL performed the genetic analysis and bioinformatics evaluations. XZ and SL conducted the clinical evaluations. SL, PL, and WZha collected clinical data and follow-up. All authors analyzed the data and approved the final article.

## References

[1] He H Guzman RE Cao D Sierra-Marquez J Yin F Fahlke C. The molecular and phenotypic spectrum of CLCN4-related epilepsy. Epilepsia. (2021) 62:1401–15. 10.1111/epi.16906 33951195

[2] Xu X Lu F Zhang L Li H Du S Tang J. Novel CLCN4 variant associated with syndromic X-linked intellectual disability in a Chinese girl: a case report. BMC Pediatr. (2021) 21:384. 10.1186/s12887-021-02860-4 34479510 PMC8414764

[3] Zhou P He N Zhang J-W Lin Z-J Wang J Yan L-M. Novel mutations and phenotypes of epilepsy-associated genes in epileptic encephalopathies. Genes Brain Behav. (2018) 17:e12456. 10.1111/gbb.12456 29314583

[4] Palmer EE Stuhlmann T Weinert S Haan E van Esch H Holvoet M. De novo and inherited mutations in the X-linked gene CLCN4 are associated with syndromic intellectual disability and behavior and seizure disorders in males and females. Mol Psychiatry. (2018) 23:222–30. 10.1038/mp.2016.135 27550844 PMC5794876

[5] Hu H Haas SA Chelly J van Esch H Raynaud M de Brouwer AP. X-exome sequencing of 405 unresolved families identifies seven novel intellectual disability genes. Mol Psychiatry. (2016) 21:133–48. 10.1038/mp.2014.193 25644381 PMC5414091

[6] Veeramah KR Johnstone L Karafet TM Wolf D Sprissler R Salogiannis J. Exome sequencing reveals new causal mutations in children with epileptic encephalopathies. Epilepsia. (2013) 54:1270–81. 10.1111/epi.12201 23647072 PMC3700577

[7] van Slegtenhorst MA Bassi MT Borsani G Wapenaar MC Ferrero GB de Conciliis L. A gene from the Xp223 region shares homology with voltage-gated chloride channels. Hum Mol Genet. (1994) 3:547–52. 10.1093/hmg/3.4.547 8069296

[8] Palmer S Perry J Ashworth A. A contravention of Ohno's law in mice. Nat Genet. (1995) 10:472–6. 10.1038/ng0895-472 7670497

[9] Di Nguyen K Yang F Kaul R Alkan C Antonellis A Friery KF. Clcn4-2 genomic structure differs between the X locus in Mus spretus and the autosomal locus in Mus musculus: AT motif enrichment on the X. Genome Res. (2011) 21:402–9. 10.1101/gr.108563.110 21282478 PMC3044854

[10] Rugarli EI Adler DA Borsani G Tsuchiya K Franco B Hauge X. Different chromosomal localization of the Clcn4 gene in Mus spretus and C57BL/6J mice. Nat Genet. (1995) 10:466–71. 10.1038/ng0895-466 7670496

[11] Jentsch TJ Pusch M. CLC chloride channels and transporters: structure, function, physiology, and disease. Physiol Rev. (2018) 98:1493–590. 10.1152/physrev.00047.2017 29845874

[12] Adler DA Rugarli EI Lingenfelter PA Tsuchiya K Poslinski D Liggitt HD. Evidence of evolutionary up-regulation of the single active X chromosome in mammals based on Clc4 expression levels in Mus spretus and Mus musculus. Proc Natl Acad Sci U S A. (1997) 94:9244–8. 10.1073/pnas.94.17.9244 9256467 PMC23138

[13] Leisle L Ludwig CF Wagner FA Jentsch TJ Stauber T. ClC-7 is a slowly voltage-gated 2Cl(-)/1H(+)-exchanger and requires Ostm1 for transport activity. EMBO J. (2011) 30:2140–52. 10.1038/emboj.2011.137 21527911 PMC3117652

[14] Jentsch TJ Günther W Pusch M Schwappach B. Properties of voltage-gated chloride channels of the ClC gene family. J Physiol. (1995) 482:19S−25. 10.1113/jphysiol.1995.sp020560 7730971 PMC1334233

[15] Suzuki T Rai T Hayama A Sohara E Suda S Itoh T. Intracellular localization of ClC chloride channels and their ability to form hetero-oligomers. J Cell Physiol. (2006) 206:792–8. 10.1002/jcp.20516 16222710

[16] Mohammad-Panah R Harrison R Dhani S Ackerley C Huan L-J Wang Y. The chloride channel ClC-4 contributes to endosomal acidification and trafficking. J Biol Chem. (2003) 278:29267–77. 10.1074/jbc.M304357200 12746443

[17] Guzman RE Bungert-Plümke S Franzen A Fahlke C. Preferential association with ClC-3 permits sorting of ClC-4 into endosomal compartments. J Biol Chem. (2017) 292:19055–65. 10.1074/jbc.M117.801951 28972156 PMC5704486

[18] Piwon N Günther W Schwake M Bösl MR Jentsch TJ. ClC-5 Cl- -channel disruption impairs endocytosis in a mouse model for Dent's disease. Nature. (2000) 408:369–73. 10.1038/35042597 11099045

[19] Mohammad-Panah R Ackerley C Rommens J Choudhury M Wang Y Bear CE. The chloride channel ClC-4 co-localizes with cystic fibrosis transmembrane conductance regulator and may mediate chloride flux across the apical membrane of intestinal epithelia. J Biol Chem. (2002) 277:566–74. 10.1074/jbc.M106968200 11675385

[20] Wang H Huo N Li F Fu S Xue Y Yang T. Osteogenic role of endosomal chloride channels in MC3T3-E1 cells. Mol Cell Biochem. (2010) 342:191–9. 10.1007/s11010-010-0483-9 20473779

[21] Ishiguro T Avila H Lin S-Y Nakamura T Yamamoto M Boyd DD. Gene trapping identifies chloride channel 4 as a novel inducer of colon cancer cell migration, invasion and metastases. Br J Cancer. (2010) 102:774–82. 10.1038/sj.bjc.6605536 20087350 PMC2837579

[22] Stauber T Weinert S Jentsch TJ. Cell biology and physiology of CLC chloride channels and transporters. Compr Physiol. (2012) 2:1701–44. 10.1002/cphy.c110038 23723021

[23] Guzman RE Grieschat M Fahlke C Alekov AK. ClC-3 is an intracellular chloride/proton exchanger with large voltage-dependent nonlinear capacitance. ACS Chem Neurosci. (2013) 4:994–1003. 10.1021/cn400032z 23509947 PMC3689194

[24] Smith AJ Lippiat JD. Voltage-dependent charge movement associated with activation of the CLC-5 2Cl-/1H+ exchanger. FASEB J. (2010) 24:3696–705. 10.1096/fj.09-150649 20501796 PMC2996913

[25] Palmer EE Pusch M Picollo A Forwood C Nguyen MH Suckow V. Functional and clinical studies reveal pathophysiological complexity of CLCN4-related neurodevelopmental condition. Mol Psychiatry. (2023) 28:668–97. 10.1038/s41380-022-01852-9 36385166 PMC9908558

[26] Guzman RE Sierra-Marquez J Bungert-Plümke S Franzen A Fahlke C. Functional characterization of CLCN4 variants associated with X-linked intellectual disability and epilepsy. Front Mol Neurosci. (2022) 15:872407. 10.3389/fnmol.2022.872407 35721313 PMC9198718

[27] Guo YX Ma HX Zhang YX Chen ZH Zhai QX. Whole-exome sequencing for identifying genetic causes of intellectual developmental disorders. Int J Gen Med. (2021) 14:1275–82. 10.2147/IJGM.S300775 33880059 PMC8053495

[28] Claes S Vogels A Holvoet M Devriendt K Raeymaekers P Cassiman JJ. Regional localization of two genes for nonspecific X-linked mental retardation to Xp22.3-p22.2 (MRX49) and Xp11.3-p11.21 (MRX50). Am J Med Genet. (1997) 73:474–9. 9415477

[29] Raynaud M Gendrot C Dessay B Moncla A Ayrault AD Moizard MP. X-linked mental retardation with neonatal hypotonia in a French family (MRX15): gene assignment to Xp11.22-Xp21.1. Am J Med Genet. (1996) 64:97–106. 8826458 10.1002/(SICI)1096-8628(19960712)64:1<97::AID-AJMG17>3.0.CO;2-N

[30] Poroca DR Pelis RM Chappe VM. ClC channels and transporters: structure, physiological functions, and implications in human chloride channelopathies. Front Pharmacol. (2017) 8:151. 10.3389/fphar.2017.00151 28386229 PMC5362633

[31] Dutzler R Campbell EB Cadene M Chait BT MacKinnon R. X-ray structure of a ClC chloride channel at 30 a reveals the molecular basis of anion selectivity. Nature. (2002) 415:287–94. 10.1038/415287a 11796999

[32] Hur J Jeong HJ Park J Jeon S. Chloride channel 4 is required for nerve growth factor-induced TrkA signaling and neurite outgrowth in PC12 cells and cortical neurons. Neuroscience. (2013) 253:389–97. 10.1016/j.neuroscience.2013.09.003 24036377

[33] Weinert S Gimber N Deuschel D Stuhlmann T Puchkov D Farsi Z et al. Uncoupling endosomal CLC chloride/proton exchange causes severe neurodegeneration. EMBO J. (2020) 39:e103358. 10.15252/embj.2019103358 32118314 PMC7196918

