# Supplementary figures and images for: Novel variants in the CLCN4 gene associated with syndromic X-linked intellectual disability
Source: Front Neurol. 2023 Sep 15;14:1096969. doi: 10.3389/fneur.2023.1096969 (PMC10542403; doi:10.3389/fneur.2023.1096969)

S Figure 1

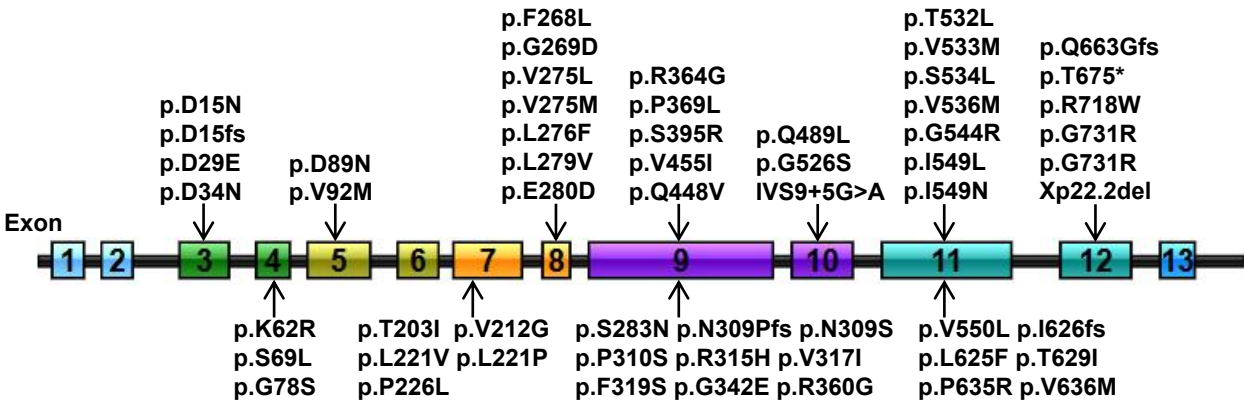

Supplement: Supplementary file 1 [file Data_Sheet_1.PDF]

S Figure 2

A

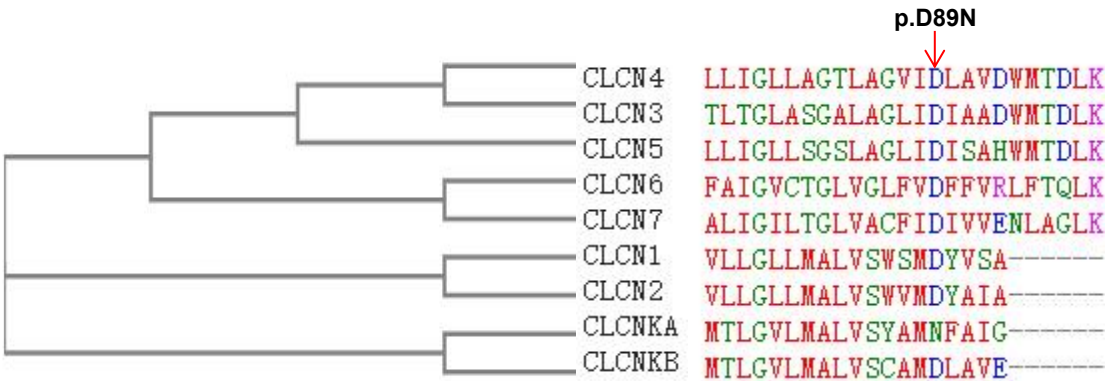

B

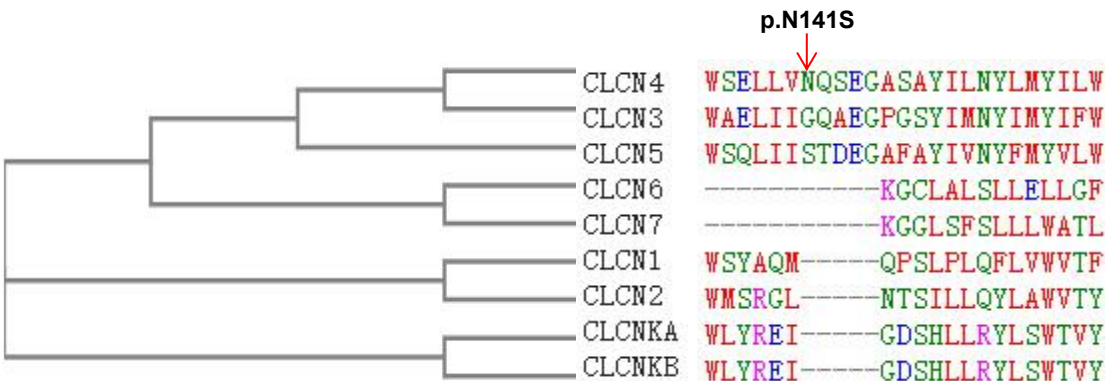

C

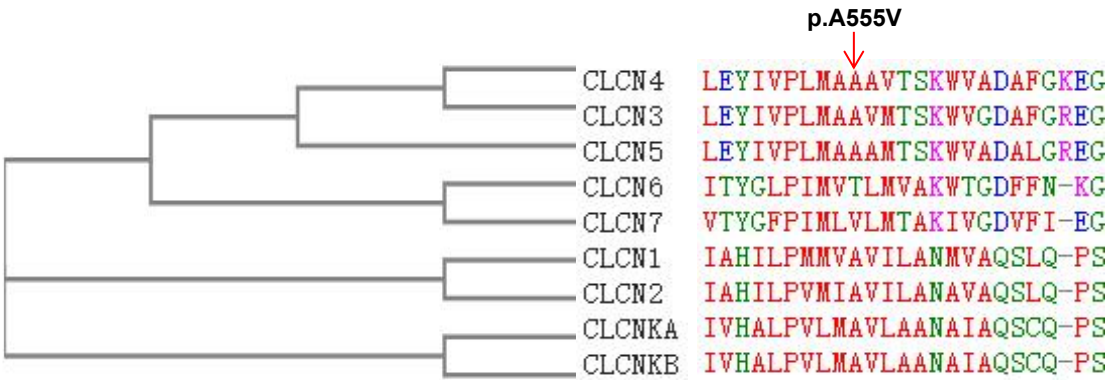

D

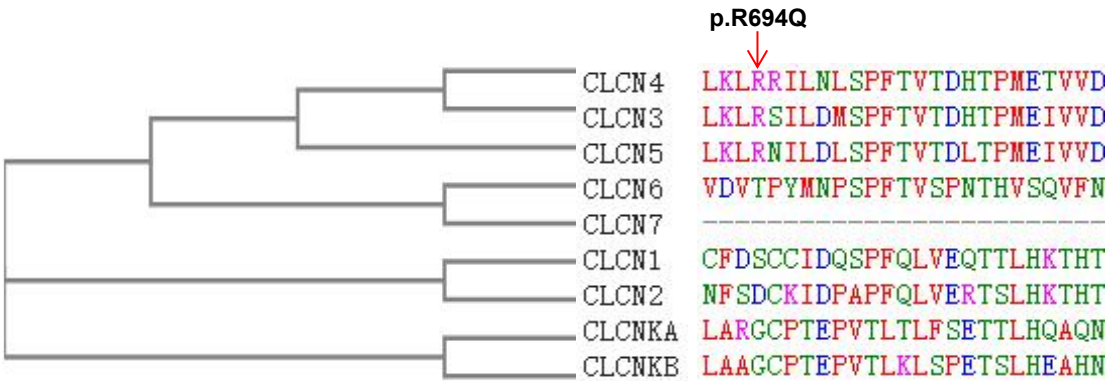

Supplement: Supplementary file 2 [file Data_Sheet_2.PDF]

S Figure 3

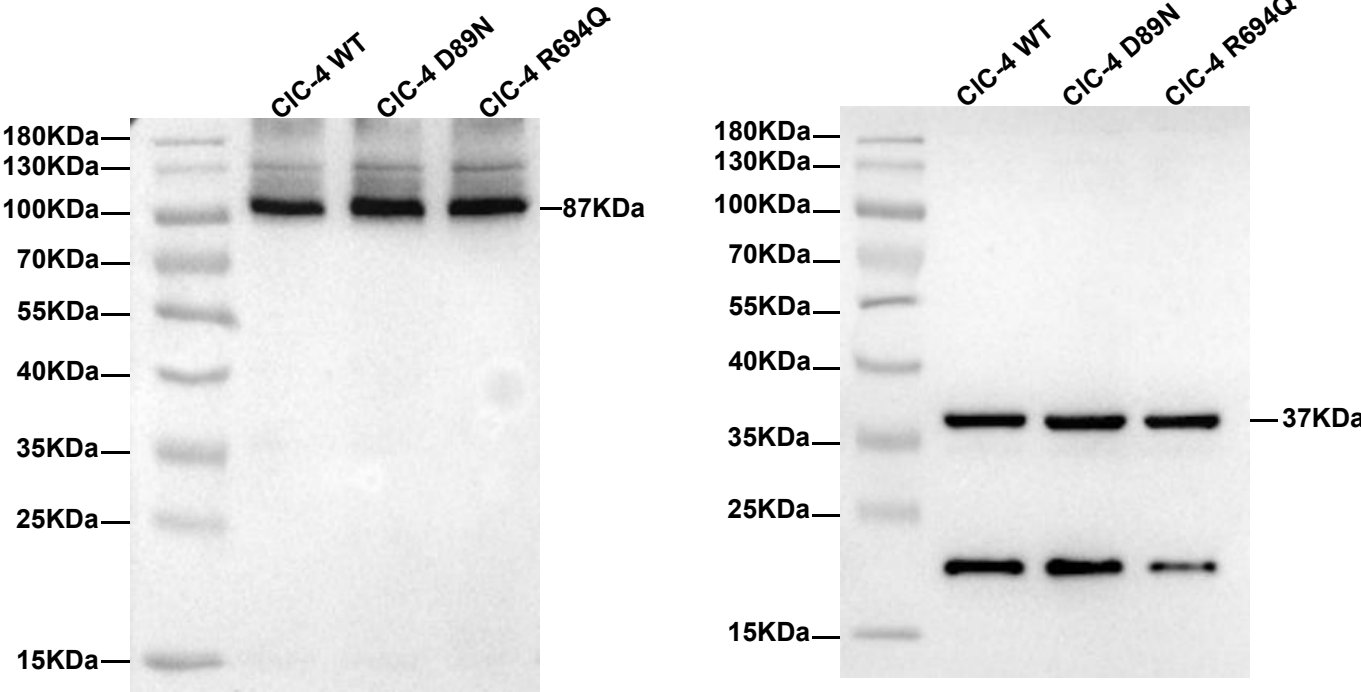

FLAG

GAPDH

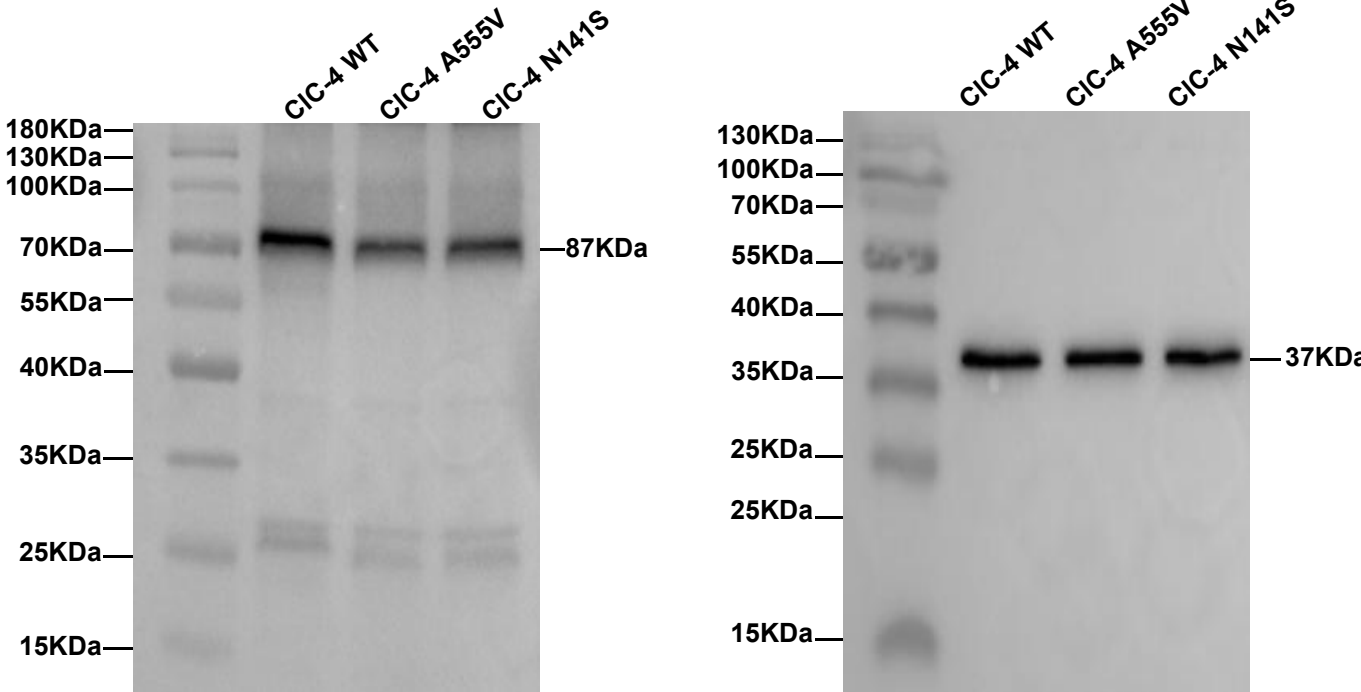

FLAG

GAPDH

Supplement: Supplementary file 3 [file Data_Sheet_3.PDF]
