# Supplementary material for: Novel variants in the CLCN4 gene associated with syndromic X-linked intellectual disability
Source: Front Neurol. 2023 Sep 15;14:1096969. doi: 10.3389/fneur.2023.1096969 (PMC10542403; doi:10.3389/fneur.2023.1096969)
Supplement: Supplementary file 4 [file Data_Sheet_4.PDF]

# Supplementary Information for

# Novel variants in the *CLCN4* gene associated with syndromic X-linked intellectual disability

**Sinan Li, Wenxin Zhang, Piao Liang, Min Zhu, Bixia Zheng, Wei Zhou, Chunli Wang*, Xiaoke Zhao***

## Methods

### Minigene assay

Genomic DNA was extracted from the peripheral blood leukocytes of our participants by a blood genomic DNA extraction kit (Tiangen, China) according to the manufacturer's instructions. Primers for PCR amplification of *CLCN4* genomic fragments were designed by web-based source Primer-Blast (F: CAGTAGATATGACTGACTAGGC; R: GTCTGCATAGTGAGCGAGAC). DNA extraction from the healthy control was performed with the complete understanding and written consent of the subject and was approved by the Ethics Committee of the Children's Hospital of Nanjing Medical University (Nanjing, China) in the study.

We generated fragments containing the target exons 10 where the splicing variant was located, and 150bp of flanking intronic regions with XhoI and BamHI restriction sites. Appropriate primers sequences were as follows: Forward:5′-accagaattctggagctcgagCAGTAGATATGACTGACTAGGC-3′; Reverse: 5′-atcaccagatatctgggatccGTCTGCATAGTGAGCGAGAC-3′. PCR fragments were purified with a PCR Purification kit (Tiangen, China). The pSPL3 minigene reporter vector including a conventional expression system with SD and SA was used as the resultant mRNA transcripts per previous research, as described in a recent study (Wang et al., 2020). Finally, the fragments were cloned into a pSPL3 vector with the XhoI and BamHI by the ClonExpressTM II One Step Cloning Kit (Vazyme Biotech Co., Ltd). All construcEach group of recombinant minigenes (empty pSPL3 control, pSPL3-E10-WT, and pSPL3-E10-MUT) was transfected to HEK239T with Lipofectamine 2000 (Invitrogen, United States) following the manufacturer's instructions. Total RNA was extracted from cells using Trizol Reagent (Takara, Japan) 24 hours after transient transfection. First-strand cDNA synthesis was reversely transcribed using the HiScript III transcriptase (Vazyme Biotech Co. Ltd) per the manufacturer's instructions. The resulting cDNA was amplified by PCR using vector-specific primers: SD (the forward primer: 5′-TCTGAGTCACCTGGACAACC-3′) and SA (the reverse primer: 5′-ATCTCAGTGGTATTTGTGAGC-3′). RT-PCR amplification for aberrant splice

transcripts, agarose gel separation, and subsequent direct Sanger sequencing was performed. ts were confirmed by bidirectional sequencing.

## Results

### Clinical Case Reports

**Patient 1** is a 3-year-7-month-old girl of nonconsanguineous Chinese parents. She was born at full-term via a cesarean section of this G1P1 mother with a birth weight of 4.3kg, without asphyxia, and with no family history of neurodevelopmental disorders. Her mother had hypothyroidism during pregnancy and was treated with levothyroxine. After birth, this girl was presented with jaundice, BS (serum bilirubin) level reached 20mg/dl, and jaundice disappeared without treatment after 1 week. She had hypertonia in infancy. Delays in her development were noted at age of 5 months, she had unsteady head control at 5 months, sat at 1 year and 4 months, and could not walk or speak at 2 years. Gesell Developmental Schedules demonstrated mild global developmental delay (GDD) at 5 months of age. She has minor dysmorphic features including right eye internal strabismus and low hairline. Physical examination at the age of 5 months showed a height of 56.6cm (< 3rd centile), a weight of 5.76kg (< 3rd centile), and a head circumference of 36.7cm (< 3rd centile). Brain MRI performed at age of 11 months showed delayed myelination. The laboratory examination was unremarkable. After comprehensive consideration of the clinical and laboratory findings, as well as her family history, we made a diagnosis of global developmental delay (GDD), and genetic analyses of the *CLCN4* gene were suggested. She was found to have a de novo heterozygous constitutive c.265 G>A (p.(Asp89Asn)) variant in *CLCN4* that was not detected in either parent.

**Patient 2** is a 3-year-1-month-old boy. He was the sixth child of healthy nonconsanguineous Chinese parents and was born full term with a normal birth weight (3400g). There was no family history of neurologic disorders. His mother once had 4 spontaneously aborted fetuses. His older sister showed normal mental and language development. At age of 2.5 years, he developed a moderate intellectual disability (ID), cannot understand instructions, and could not speak any words or use signs, only syllables. He lacked visual contact and showed behavioral abnormalities with an outburst of temper tantrums, crying, and hyperactivity behavior. He had stereotypies and repetitive actions, always repeatedly putting on and taking off socks, dragging the door, and repeatedly looking at his hands at home. Physical examination at 2.5 years showed a height of 90.1cm (< 50th centile), a weight of 11.9kg (< 10th centile), and a head circumference of 48.4cm (< 50th centile). Ophthalmology and hearing examinations showed normal. And he was also evaluated by the Autistic Behavior Checklist (ABC) (ABC: score <50) and Developmental Screen Test (DST): Developmental quotient (DQ) score <50, and Mental Index (MI) score <50. Brain MRI is absent. Other neurometabolic investigations were no particular. He was found to have a hemizygous constitutive c.422A>G (p.(Asn141Ser)) variant in *CLCN4* that was inherited from his unaffected mother.

**Patient 3** is a 3-year-6-month-old girl. She was born at 36-week via a cesarean section with a birth weight of 2.55kg and a length of 44cm and the first child of nonconsanguineous Chinese parents, without asphyxia, with no family history of neurological disorders. Her mother had less amniotic fluid in the late pregnancy, and during the pregnancy, she was kept in the local hospital for 10 days due to hypertension and hyperglycemia. Hypertonia and delayed psychomotor development became obvious at age of 7 months. She could not sit or crawl. At the age of 11 months, her height was 68.8 cm (< 3rd centile), weight was 7.8 kg (< 3rd centile) and head circumference was 40.6 cm (< 3rd centile). She was evaluated by the DST: DQ score <45, and MI score <50. At the age of 2 years, her height was 80.3 cm (< 10th centile), weight was 9.8 kg (< 3rd centile) and head circumference was 42.3 cm (< 3rd centile). After regular rehabilitation training and physiotherapy, the child will walk at the age of 2 years. Brain MRI was performed that widened bilateral frontal-temporal extra brain space and enlarged the left ventricle. She was found to have a de novo heterozygous constitutive c.1644C>T (p.(Ala555Val)) variant in *CLCN4* that was not detected in either parent.

**Patient 4** is a 5-year-11-month-old girl. She was born at full term to non-consanguineous Chinese parents with a birth weight of 3.78kg. She had mild neonatal jaundice which did not require treatment and neonatal intracranial hemorrhage was treated in the hospital for 3 days. At 1 year old, she could not roll over, could not sit, and was unstable sitting alone at 1 year and 1 month. She could not walk alone or speak at 4 years and 7 months until now. And she developed difficulty swallowing, had slight limb hypertonia, and had occasional involuntary shaking, however, she had no obvious history of seizures. Brain MRI was performed that brain dysplasia: small cranial volume, slightly thin the posterior of the corpus callosum, and widened ventricles. She was found to have a de novo heterozygous constitutive c.1644C>T (p.(Ala555Val)) variant in *CLCN4* that was not detected in either parent.

**Patient 5** is a 5-year-8-month-old boy, born at the normal term of gestation without asphyxia after an uneventful pregnancy, who is the first child of healthy unrelated Chinese parents, and no genetic diseases were present in the family history. Delays in his development were noted in the first year of life. He started walking at the age of 14 months. He developed ID and showed marked language delay. He was non-verbal at the age of 2 years and 8 months. He was not able to speak any meaningful words, but regression was not reported. He could not recognize facial features, indicate size, numbers, and colors, and could merely finish simple instructions occasionally. He showed behavioral abnormalities with an outburst of temper tantrums, excessive crying, stereotyped repetitive and hyperactivity behavior. He lacked concentration and did not have infantile hypotonia. Physical examination at the age of 2 years and 9 months showed height was 92.5cm (< 50th centile), weight was 12.96kg (< 50th centile) and head circumference was 48.4 cm (< 50th centile). Neuropathy revealed that he had poor visual and auditory facial reflexes. He had no formal behavioral or psychiatric diagnoses, and no obvious craniofacial dysmorphism. His brain MRI was normal. Plasma electrolytes, plasma glucose, full blood examination, ammonia, liver

function tests, renal function, plasma amino acids and acylcarnitine profile, urine amino and organic acids were normal. The genetic analyses revealed a hemizygous missense variant c.2081G>A (p.(Arg694Gln)) in the *CLCN4* gene. His mother is heterozygotes and clinically unaffected.

**Patient 6** is a 2-year-4-month-old boy born after uncomplicated pregnancy and delivery from healthy nonconsanguineous parents of Chinese origin. He had septicemia in infancy. Aged 1 year he could stand and walk with no support. Language development has been a particular area of concern and he is non-verbal at the age of 2 years. He was not able to speak any meaningful words, only syllables at the age of 2 years and 4 months. At the same age, he could recognize facial features occasionally, and could not indicate colors, size, or numbers. He was unable to complete simple instructions and answer any questions. He showed abnormal social interaction, poor eye contact, and was not interested in his surroundings. Brain MRI, performed at the age of 2 years and 4 months, revealed a possible right temporal arachnoid cyst and slightly wide left temporal subcranial plate gap. His father had perforating palm, but no intellectual disability. He was found to have a maternally inherited splice site variant c.1390-12(IVS9) T>G in *CLCN4* that was inherited from his unaffected mother.

## Figure legends

**S Figure 1. Mapping of all *CLCN*4 variants functionally published to date.**

Schematic of the *CLCN4* gene with position of variants published to date shown above and below the schematic. The boxes indicate exon, "1 to 13" indicate exon number.

**S Figure 2. Evolutionary conservation of human CLCs family.**

A-D parts respectively represent CLCs phylogram and multiple sequence alignment of our four missense variants.

**S Figure 3. The whole gel of western blot experiment.**

The legend includes the molecular weights of the marker bands, the name of the target protein, and the antibody used for detection.

**S Table 2 In silico analysis of the *CLCN4* Variants.**

| Transcript ID | Variant | Polyphen2_HDIV | Polyphen2_HVAR | SIFT | Provean | Mutation Taster | CADD |
|---|---|---|---|---|---|---|---|
| **NM_001830.4** | c.265G>A/(p.(Asp89Asn)) | 0.999, D | 0.918 | 0.03 | -4.777 (Deleterious) | Disease causing | 24.7 |
| | c.422A>G/(p.(Asn141Ser)) | 0.000 | 0.001 | 0.51 | 0.279 (Neutral) | Polymorphism | 19.91 |
| | c.1644C>T/(p.(Ala555Val)) | 0.965, D | 0.627, P | 0.88 | -3.302 (Deleterious) | Disease causing | 23.1 |
| | c.2081G>A/(p.(Arg694Gln)) | 0.998, D | 0.617, P | 0.24 | -2.461(Neutral) | Disease causing | 24.8 |

| Variant | MaxEntScan | dbscSNV | GTAG | spliceAI |
|---|---|---|---|---|
| c.1390-12(IVS9)T>G | Deleterious (8.95->6.06) | Deleterious (0.9993 │ 0.9240) | -- | -- |

Abbreviations: PolyPhen-2, Polymorphism Phenotyping v2; SIFT, Sorting Intolerant from Tolerant; PROVEAN, Protein Variation Analysis Effector; CADD, Combined Annotation Dependent Depletion.

Note: Polyphen2_HDIV: D means probably damaging (pp2_hdiv ≥ 0.957); Polyphen2_HVAR: P means possibly damaging (0.447 ≤ pp2_hvar ≤ 0.909); SIFT: D means deleterious (sift ≤ 0.05); PROVEAN: Deleterious < -2.5; CADD: variant is regarded as deleterious when its score greater than 15.
